# The Dixie Doctor

**Published:** 1890-09

**Authors:** 


THAT bright new Southern journal, *The Dixie Doctor*, says :

Many of our exchanges think it no harm to cull from our columns without credit. It is remarkable how tough and elastic their consciences are, and how innocently they steal the result of much laborious work and time spent in condensing newsy matter. One clip of the shears and they take lazily what they cannot or will not do for themselves. We would name some of these transgressors, but do not wish to give them what they seek—free advertisement.

True ; but will the good *Dixie Doctor* tell us where he culled the note on the Pronunciation of Gynecology in his July number?

